# Associations Between Alzheimer’s Disease and Obesity: Clinical Trials of NA‐831 for AD and NA‐931 for Obesity

**DOI:** 10.1002/alz.095715

**Published:** 2025-01-09

**Authors:** Lloyd Tran, Zung Vu Tran, Fern Vu

**Affiliations:** ^1^ Biomed Industries, Inc., San Jose, CA USA

## Abstract

**Background:**

NA‐831 is a new drug candidate, exhibiting neuroprotection, neurogenesis and memory enhancing properties for the treatment of Alzheimer’s Disease (AD). NA‐931 is an analog of NA‐831, regulating the homeostasis of the triple: IGF‐1, GLP‐1 and GIP functions for obesity.

**Method:**

A randomized clinical trial of NA‐831 was performed in 112 participants with mild and moderate AD, half received the drugs and half received placebo. The patients with MCI received 10 mg of NA‐831 and patients with mild and moderate AD received 30 mg of NA‐831 or placebo orally per day. Subjects with MCI to meet the NIA‐AA core clinical criteria, CDR score of 0.5 and a Memory Box score of 0.5 or greater at Screening and Baseline. MMSE score ≥22. Subjects with mild & moderate AD to meet the NIA‐AA core clinical criteria. MMSE: 17‐21.

Animal Studies of NA‐931: A 14‐day study in diet‐induced obesity DIO‐NASH mice was conducted to evaluate the treatment of NA‐931 on obesity.

**Result:**

NA‐831 showed a significant improvement for patients with mild and moderate AD with the ADAS‐Cog‐13 score change of an average of 4.1 as compared to the placebo after 24 weeks of treatment (p = 0.001; ITT). CIBIC‐Plus showed 78% patients improved (p = 0.01; ITT). mNA‐831 was well‐tolerated at 30 mg/day. It was observed that 66% (12 of 18) patients having diabetes amongst 56 patients on the NA‐831 drug treatment reported a loss of 17‐23% of body weight over 6 months. Only 4 of 7% of 56 patients on placebo reported a loss of 3‐5% over 6 months. There were no serious adverse events observed.

The study in DIO‐NASH mice had demonstrated that treatment with NA‐931 resulted in significant reductions in body weight up to 26% (p<0.0001), as well as reductions in plasma glucose and plasma triglycerides up to 23% and 34%, respectively (p<0.003 for each). NA‐931can reduce the body mass index, without causing the muscle loss.

**Conclusion:**

An association of Alzheimer’s disease and diabetes obesity has been suggested with clinical results of NA‐831 and NA‐931. However, whether this association is causal requires further evaluation.

